# Mitochondria-targeted esculetin alleviates mitochondrial dysfunction by AMPK-mediated nitric oxide and SIRT3 regulation in endothelial cells: potential implications in atherosclerosis

**DOI:** 10.1038/srep24108

**Published:** 2016-04-11

**Authors:** Santosh Karnewar, Sathish Babu Vasamsetti, Raja Gopoju, Anantha Koteswararao Kanugula, Sai Krishna Ganji, Sripadi Prabhakar, Nandini Rangaraj, Nitin Tupperwar, Jerald Mahesh Kumar, Srigiridhar Kotamraju

**Affiliations:** 1 Centre for Chemical Biology, CSIR-Indian Institute of Chemical Technology, Uppal Road, Hyderabad, 500007, India; 2 Academy of Scientific and Innovative Research, Training and Development Complex, Chennai, India; 3 National Centre for Mass Spectrometry, Indian Institute of Chemical Technology, Hyderabad, 500007, India; 4 CSIR-Centre for Cellular and Molecular Biology, Uppal Road, Hyderabad, 500007, India

## Abstract

Mitochondria-targeted compounds are emerging as a new class of drugs that can potentially alter the pathophysiology of those diseases where mitochondrial dysfunction plays a critical role. We have synthesized a novel mitochondria-targeted esculetin (Mito-Esc) with an aim to investigate its effect during oxidative stress-induced endothelial cell death and angiotensin (Ang)-II-induced atherosclerosis in ApoE^−/−^ mice. Mito-Esc but not natural esculetin treatment significantly inhibited H2O2- and Ang-II-induced cell death in human aortic endothelial cells by enhancing NO production via AMPK-mediated eNOS phosphorylation. While L-NAME (NOS inhibitor) significantly abrogated Mito-Esc-mediated protective effects, Compound c (inhibitor of AMPK) significantly decreased Mito-Esc-mediated increase in NO production. Notably, Mito-Esc promoted mitochondrial biogenesis by enhancing SIRT3 expression through AMPK activation; and restored H2O2-induced inhibition of mitochondrial respiration. siSIRT3 treatment not only completely reversed Mito-Esc-mediated mitochondrial biogenetic marker expressions but also caused endothelial cell death. Furthermore, Mito-Esc administration to ApoE^−/−^ mice greatly alleviated Ang-II-induced atheromatous plaque formation, monocyte infiltration and serum pro-inflammatory cytokines levels. We conclude that Mito-Esc is preferentially taken up by the mitochondria and preserves endothelial cell survival during oxidative stress by modulating NO generation via AMPK. Also, Mito-Esc-induced SIRT3 plays a pivotal role in mediating mitochondrial biogenesis and perhaps contributes to its anti-atherogenic effects.

Atherosclerosis is an excessive inflammatory/proliferative response of the vascular wall to various forms of injury^1^,^2^. It has been suggested that, during inflammation, reactive oxygen species (ROS)- and reactive nitrogen species (RNS)-induced endothelial cell damage represent an important primary event in the process of atherosclerotic lesion formation^3^,^4^. The resulting oxidative and nitrosative stress impairs the critical balance of the availability of endothelium-derived nitric oxide, in turn, promoting the proinflammatory signaling events ultimately leading to the plaque formation. Atherosclerosis initiating events may be different under different conditions; however endothelial dysfunction is known to be one of the major initiating events^5^.

Increased mitochondrial oxidative damage is a major feature of most age-related human diseases including atherosclerosis and atypical electron leakage from mitochondria in the respiratory chain in oxidant-stressed cells triggers the formation of ROS in mitochondria leading to altered behavior of the cell/cell death^6^. Earlier studies have linked excess generation of ROS with vascular lesion formation and functional defects^3^,^4^,^7^. More so, a role for mitochondria-derived ROS in atherogenesis is supported by links between common risk factors for coronary artery disease and increased levels of ROS^8^. Mitochondrial ROS is increased in response to many atherosclerosis inducers including hyperglycemia, triglycerides and ox-LDL^9^,^10^,^11^. Aortic samples from atherosclerotic patients had greater mitochondrial DNA (mtDNA) damage than nonatherosclerotic aortic samples from age-matched transplant donors^12^. Even though endothelial cells have low mitochondria content, mitochondrial dynamics acts as a prime orchestrator of endothelial homeostasis under normal conditions and an impairment of mitochondrial function because of excess ROS production would lead to endothelial dysfunction resulting in diverse vascular disorders^13^. Exposure of endothelial cells to free fatty acids, a common feature seen in patients with metabolic syndrome increases mitochondrial ROS^14^.

Therefore keeping given the involvement of mitochondrial ROS in causing endothelial dysfunction leading to the enhancement of vascular diseases, it would be ideal to either counteract mitochondrial ROS by targeting ROS scavengers specifically to the site of action or it is perhaps beneficial to enhance mitochondrial biogenesis to reduce the burden during stress-induced mitochondrial dysfunctions. One of the limiting factors with antioxidant therapy in the treatment of mitochondrial diseases has been the failure to enhance antioxidant levels in mitochondria. Recently, there was a breakthrough in mitochondrial targeting of antioxidants^15^,^16^. Antioxidant molecules were covalently coupled to a triphenylphosphonium cation (TPP^+^), and these compounds were preferentially taken up by mitochondria^15^. The lipophilic cations easily pervade through the lipid bilayers and subsequently build up several hundred-fold within mitochondria because of a large mitochondrial membrane potential^15^. This strategy not only reduces the concentration of the molecule that is being employed to scavenge ROS, but also reduces the nonspecific effects of the molecule if it were to be used at high concentrations to elicit a similar effect.

Coumarins consist of a group of phenolic compounds widely distributed in natural plants, and they have recently attracted much attention because of their wider pharmacological activities^17^,^18^. Of these, esculetin (6, 7-dihydroxycoumarin) has been shown to be a lipoxygenase inhibitor. It inhibits the production of leukotrienes and hydroxyeicosatetraenoic acid through the lipoxygenase pathway^19^. More recently, esculetin has been reported to inhibit oxidative damage induced by tertbutyl hydroperoxide in rat liver^20^. Esculetin protects against cytotoxicity induced by linoleic acid hydroperoxide in HUVEC cells, and the radical scavenging ability of esculetin was confirmed by electron paramagnetic resonance spectroscopy^21^. However, as coumarins may have reduced bioavailability *in vivo* and do not significantly accumulate within mitochondria, their effectiveness remained limited and because of this, they may have to be employed at higher concentrations to scavenge mitochondrial ROS. In the present study,we have used lipophilic cation (TPP^+^) to preferentially target esculetin to mitochondria and show that mitochondria-targeted esculetin (Mito-Esc) protects against oxidant-induced endothelial cell death via nitric oxide and AMPKα-dependent pathways at far below concentrations than that was reported earlier with natural esculetin. Also, Mito-Esc significantly enhanced SIRT3 expression, a mitochondrial histone deacetylase, shown to be involved in mitochondrial biogenesis. In addition, we report that Mito-Esc but not natural esculetin significantly inhibits angiotensin (Ang-II)-induced atheromatous plaque formation in ApoE^−/−^ mice.

## Results

### Mitochondria-targeted esculetin (Mito-Esc) but not natural esculetin abrogates oxidant-induced cell death in human aortic endothelial cells (HAEC)

**Figure 1:**
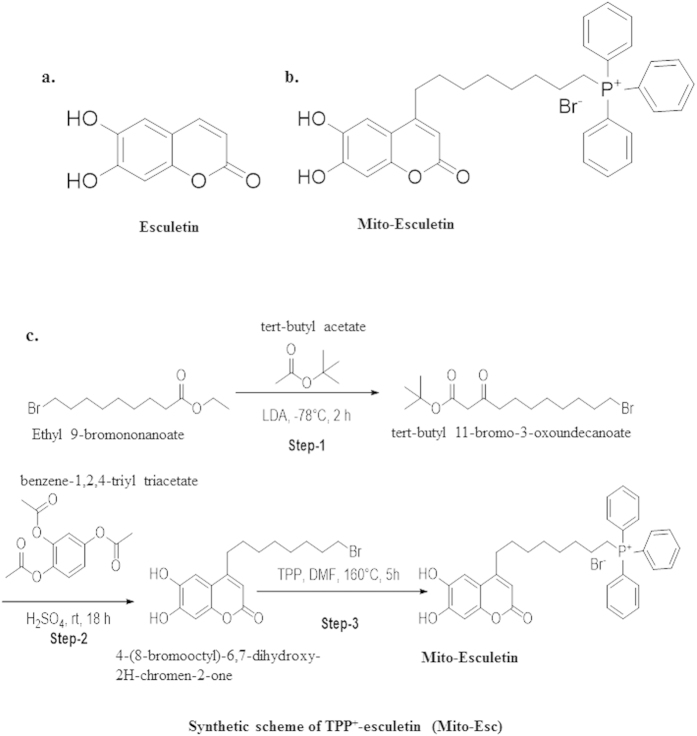
Structures of esculetin (**a**), Mito-Esculetin (**b**) and synthetic scheme of Mito-Esculetin (**c**).

**Figure 2:**
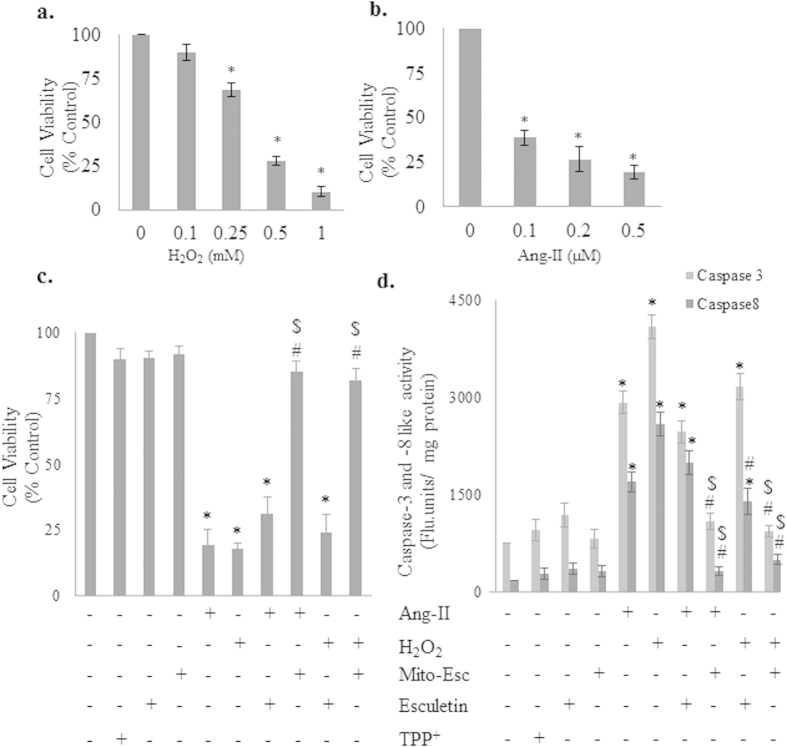
Effect of mitochondria-targeted esculetin (Mito-Esc) and parent esculetin on oxidative stress-induced endothelial cell death and apoptosis. (**a**,**b**) Human aortic endothelial cells (HAEC) were treated with H_2_O_2_ (0.1–1.0 mM) and Ang-II (0.1–0.5 μM) respectively for 24 h and cell viability was measured by trypan blue assay. (**c**) HAEC were pretreated with esculetin (2.5 μM), Mito-Esc (2.5 μM), TPP^+^ (2.5 μM) for 2 h followed by H_2_O_2_ (500 μM) or Ang-II (0.5 μM) for 24 h and cell viability was measured by trypan blue assay. (**d**) Is same as *c* except that caspase-3 and -8 activities were measured. *significantly different (p < 0.05) compared to untreated conditions. ^#^Significantly different (p < 0.05) compared to Ang-II or H_2_O_2_ treated condition. ^$^Significantly different (p < 0.05) compared to Esculetin + H_2_O_2_ treated condition. Results indicated were average of three independent experiments.

In this study we have synthesized a novel mitochondria-targeted esculetin (Mito-Esc) by covalently coupling esculetin with a lipophilic triphenylphosphonium cation tagged with octenyl carbon chain [TPP^+^] (Fig. 1) and initially compared the efficacy of Mito-Esc over esculetin during oxidant-induced endothelial cell death. HAEC were incubated with various concentrations of either H_2_O_2_ or angiotensin (Ang)-II for 24 h, and cell viability was measured by trypan blue dye exclusion method. Both H_2_O_2_ and Ang-II dose-dependentlycaused cell death in HAEC (Fig. 2a,b). Next, the effects of Mito-Esc, as well as the natural esculetin on Ang-II and H2O2-induced endothelial cell death, were studied. For this, cells were pretreated for 2 h with either Mito-Esc or esculetin before they were incubated with either H_2_O_2_ or Ang-II. Interestingly, Mito-Esc but not natural esculetin significantly inhibited oxidant-induced endothelial cell death (Fig. 2c). Under these conditions, TPP^+^ alone did not show any appreciable cytotoxic/cytoprotective effect in HAEC (Fig. 2c). Thereby, indicating that the observed protective effect with Mito-Esc was not because of the TPP^+^ side chain coupled to esculetin. Next, to confirm that H_2_O_2_ and Ang-II caused an apoptotic cell death, caspase-3 and -8 activities were measured. Mito-Esc-pretreated cells were markedly resistant to H_2_O_2_- or Ang-II-induced caspase activation, whereas treatment with natural esculetin elicited the marginal effect on caspase-3 and -8 activation (Fig. 2d). These observations are consistent with the cell death results.

### Mito-Esc treatment decomposes Ang-II-induced H_2_O_2_ generation, preserves oxidant-mediated depolarization of mitochondrial membrane potential and inhibits peroxide-induced mitochondrial superoxide production

**Figure 3:**
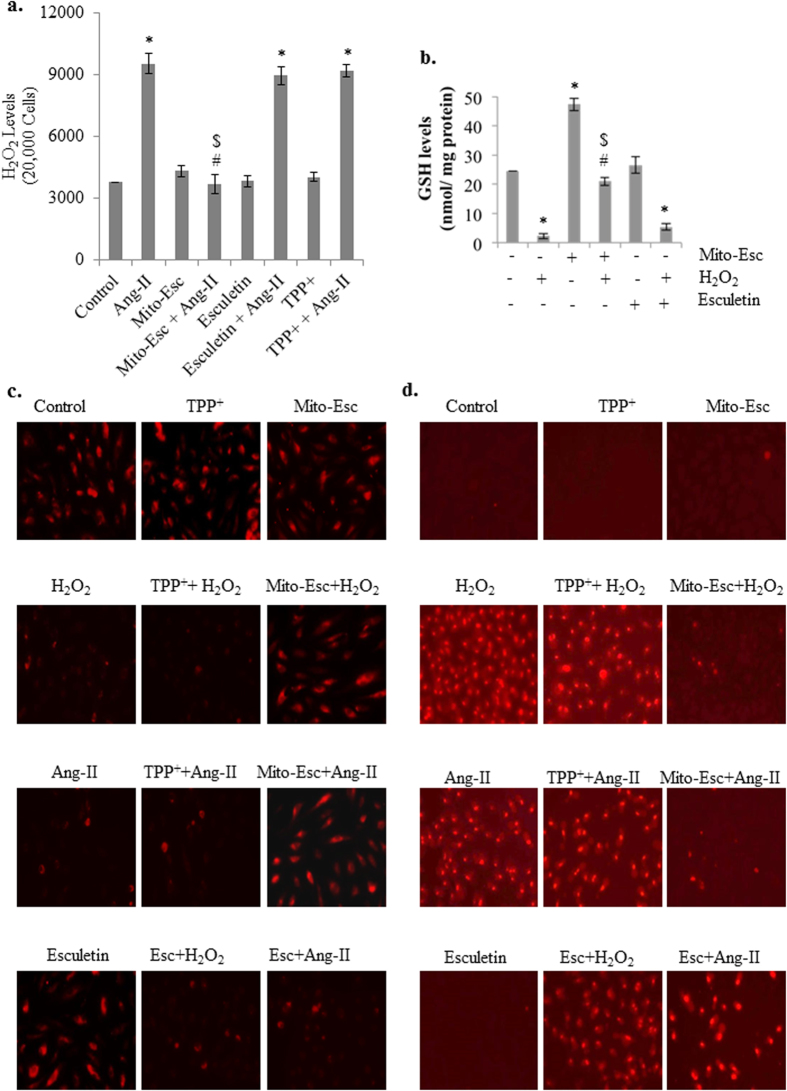
Mito-Esc restores oxidant-induced depletion of GSH levels, dysregulation of mitochondrial transmembrane potential and mitochondrial superoxide generation in endothelial cells. (**a**) HAECs were pretreated with Mito-Esc (2.5 μM) or esculetin (2.5 μM) or TPP^+^ (2.5 μM) for 2 h before the addition of Ang-II (0.5 μM) for 24 h and H_2_O_2_ levels were measured by the Amplex red assay. (**b**) Cells were pretreated with Mito-Esc or esculetin for 8 h, and GSH levels were measured as described in methods section. (**c**) HAEC were pretreated with Mito-Esc for 2 h before H_2_O_2_ (500 μM) was added for 8 h, and mitochondrial transmembrane potential was measured using TMRE fluorescence as described in methods. (**d**) Cells were treated with H_2_O_2_ or Ang-II in the presence or absence of either Mito-Esc or esculetin or TPP^+^ for 8 h and mitochondrial superoxide generation was measured by Mito-SOX dye as described in Methods section. *Significantly different (p < 0.05) compared to untreated conditions. ^#^Significantly different (p < 0.05) compared to Ang-II or H_2_O_2_ treated condition. ^$^Significantly different (p < 0.05) compared to Esculetin + H_2_O_2_ treated condition. Results indicated were average of three independent experiments.

Ang-II is known to increase oxidative stress through increased production of H_2_O_2_ 22. To see the effect of Mito-Esc in altering Ang-II-induced H_2_O_2_ production, HAEC were treated with Ang-II (500 nM) in the presence or absence of Mito-Esc (2.5 μM) for 16 h and H_2_O_2_ production was measured by Amplex red assay^23^. Ang-II treatment significantly increased H_2_O_2_ generation by around 2.7 fold compared to untreated conditions (Fig. 3a). Interestingly, Mito-Esc co-treatment completely reversed H_2_O_2_ production to control conditions (Fig. 3a). In conjunction with this, Mito-Esc but not esculetin treatment significantly restored H_2_O_2_-induced depletion of GSH levels (Fig. 3b). It is to be noted that Mito-Esc treatment alone greatly increased GSH levels (Fig. 3b). Thereby suggesting that, Ang-II-induced cytotoxicity in HAEC involves oxidative stress and that co-incubation with Mito-Esc greatly attenuates Ang-II-induced cell death possibly by enhancing GSH levels. Further, we assessed the effect of Mito-Esc on H_2_O_2_-induced mitochondrial membrane depolarization. HAEC were treated with H_2_O_2_ (500 μM) in the presence or absence of Mito-Esc for 8 h, and mitochondrial membrane potential was measured using Tetramethylrhodamine, ethyl ester (TMRE). TMRE fluorescent dye is taken up only by active mitochondria of healthy cells and exhibits red fluorescence. In agreement with the results shown in Fig. 3a,b, Mito-Esc but not esculetin significantly rescued H_2_O_2_- and Ang-II-mediated mitochondrial membrane depolarization (Fig. 3c). Under similar conditions, mitochondrial superoxide production was measured by the oxidation of mitochondria-targeted hydroethidine (Mito-SOX) which reportedly measures mitochondrial superoxide generation. A significant increase in the red fluorescence in H_2_O_2_ and Ang-II treated HAEC was observed indicative of enhanced mitochondrial superoxide production (Fig. 3c). Under these conditions, however, co-incubation of cells with Mito-Esc but not esculetin greatly inhibited mitochondrial superoxide staining, and incubation of cells with TPP^+^ either alone or with any of the above-treated conditions had no effect on mitochondrial superoxide generation (Fig. 3d). These results signify that Mito-Esc protects endothelial cells during oxidant stress.

### Mito-Esc potentiates nitric oxide generation via increased eNOS phosphorylation in HAEC: Effect of NOS inhibitor on Mito-Esc-mediated inhibition of cell death during oxidative stress

**Figure 4:**
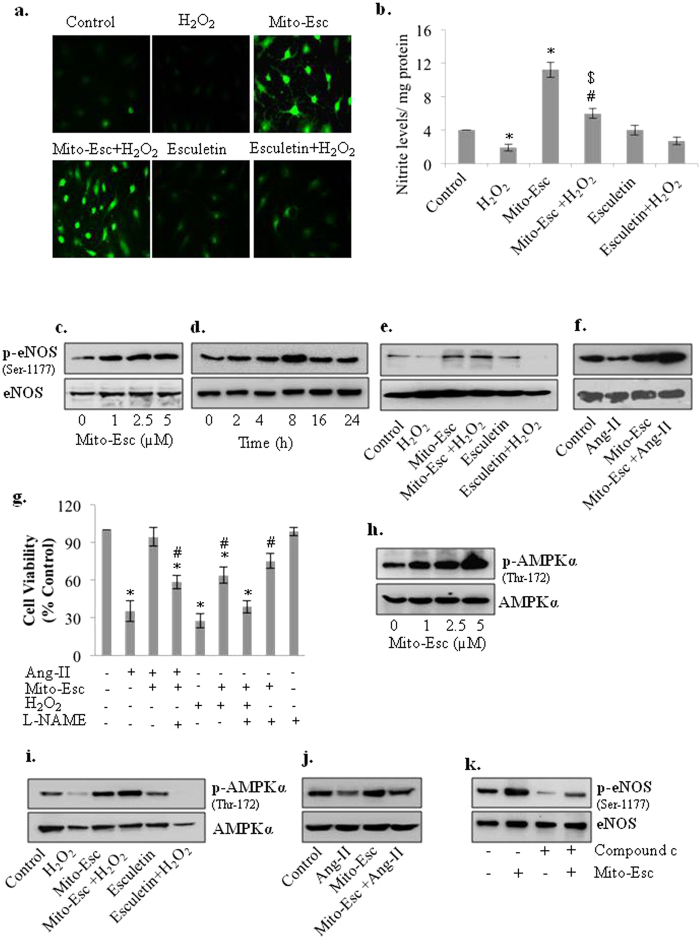
Mito-Esc restores H_2_O_2_-induced inhibition of AMPKα and eNOS phosphorylation in HAEC. (**a**) HAECs were pretreated with either Mito-Esc (2.5 μM) or esculetin (2.5 μM) for 2 h before H_2_O_2_ (500 μM) was added for 8 h and nitric oxide levels were measured by DAF-2DA fluorescence as described in methods. (**b**) Same as *a,* except that nitrite levels were measured. (**c–f**) HAEC were treated with various conditions as indicated for 8 h after which AMPKα, eNOS and phospho-eNOS (Ser-1177) protein levels were measured by western blot analysis. (**g**) HAEC were pretreated with esculetin, Mito-Esc, L-NAME (2 mM) for 2 h before the addition of Ang-II or H_2_O_2_ for 24 h and cell viability was measured by trypan blue assay. (**h–j**) Same as *c, e, f* except that, phospho-AMPKα (Thr-172) and AMPKα, protein levels were measured by immuno blotting. AMPKα (**k**) HAEC were treated with Mito-Esc in presence or absence of Compound c for 8 h and eNOS and p-eNOS (Ser-1177) protein levels were measured by Western blot analysis. The data shown represents from three independent experiments *significantly different (p < 0.05) compared to untreated conditions. ^#^Significantly different (p < 0.05) compared to Ang-II or H_2_O_2_ treated condition. ^$^Significantly different (p < 0.05) compared to Esculetin + H_2_O_2_ treated condition.

Nitric oxide plays a pivotal role in maintaining vascular tone while endothelial dysfunction as a result of impairment of mitochondrial function has been shown to precede the development of cardiovascular diseases^24^. To gain mechanistic insights on Mito-Esc-mediated protection during oxidant-induced endothelial cell death, we hypothesized that Mito-Esc may augment intracellular nitric oxide generation. To study this, HAEC were treated with both Mito-Esc and natural esculetin in the presence or absence of H_2_O_2_ for 8 h and nitric oxide levels were monitored by DAF-2 derived green fluorescence and nitrite production^25^,^26^. Intriguingly, Mito-Esc alone but not natural esculetin greatly enhanced DAF-2 fluorescence and nitrite levels. (Fig. 4a,b and Supplementary Fig. S1). There by indicating that incubation of endothelial cells with Mito-Esc causes an increase in NO production. Also, while Mito-Esc significantly rescued, natural esculetin did not show any noticeable effect on H_2_O_2_-mediated depletion of NO production (Fig. 4a,b and Supplementary Fig. S1). Next, we investigated the possible role of eNOS in mediating the Mito-Esc-induced NO generation in HAEC. Mito-Esc dose-dependently increased the phosphorylation of eNOS at Ser-1177 (Fig. 4c and Supplementary Fig. S1) and this increase in eNOS phosphorylation by Mito-Esc sustained till 24 h (Fig. 4d). To further substantiate these results, eNOS phosphorylation was measured with either H_2_O_2_ or Ang-II treatment for 8 h in cells pretreated with Mito-Esc or natural esculetin. Both H_2_O_2_ (500 μM) and Ang-II (0.5 μM) caused a reduction in Phospho-eNOS levels (Fig. 4e,f and Supplementary Fig. S1). In contrast, Mito-Esc but not natural esculetin co-treatment caused cells resistant to oxidant-mediated decrease in eNOS-phosphorylation (Fig. 4e,f). Under these conditions, however, incubation of cells with L-NAME (NOS inhibitor) significantly abrogated Mito-Esc-mediated cytoprotective effects (Fig. 4g). Taken together, these results suggest that Mito-Esc-mediated increase in nitric oxide generation via increased phosphorylation of eNOS, is in part, responsible for maintaining endothelial cell viability during oxidative stress.

### Mito-Esc-mediated AMPK activation is responsible for eNOS phosphorylation and NO generation

Previously, it was shown that AMPKα co-immunoprecipitates with cardiac endothelial NO synthase and phosphorylates Ser-1177 in the presence of Ca^2+^-calmodulin to activate eNOS both *in vitro* and during ischemia in rat hearts^27^. To test whether the Mito-Esc-mediated increase in eNOS phosphorylation was due to AMPK activation, HAEC were treated with various concentrations of Mito-Esc for 8 h and AMPKα phosphorylation (Thr-172) levels were measured. Mito-Esc lead to a dose-dependent increase in phospho-AMPKα (Fig. 4h and supplementary Fig. S1). However, incubation of cells with Mito-Esc but not with natural esculetin for 8 h significantly rescued H_2_O_2_-induced depletion of AMPKα phosphorylation (Fig. 4i and Supplementary Fig. S1). Similar results were obtained with Ang-II treatment, where it was found that Ang-II treatment significantly down-regulated phospho-AMPKα levels and that co-incubation with Mito-Esc made cells resistant to Ang-II-mediated inhibition of phospho-AMPKα (Fig. 4j and Supplementary Fig. S1). Next, to see if Mito-Esc-mediated AMPKα activation was responsible for the enhancement of eNOS phosphorylation, cells were treated with Compound c (AMPK inhibitor) in the presence or absence of Mito-Esc for 8 h and found that Compound c completely abrogated Mito-Esc-mediated increase in eNOS phosphorylation (Fig. 4k and Supplementary Fig. S1). These results suggest that Mito-Esc-induced NO generation is possibly regulated by AMPKα activation that, in turn, inhibited endothelial cell death during oxidative stress.

### Oxidative stress-induced deregulation of mitochondrial biogenesis is rescued in the presence of Mito-Esc

**Figure 5:**
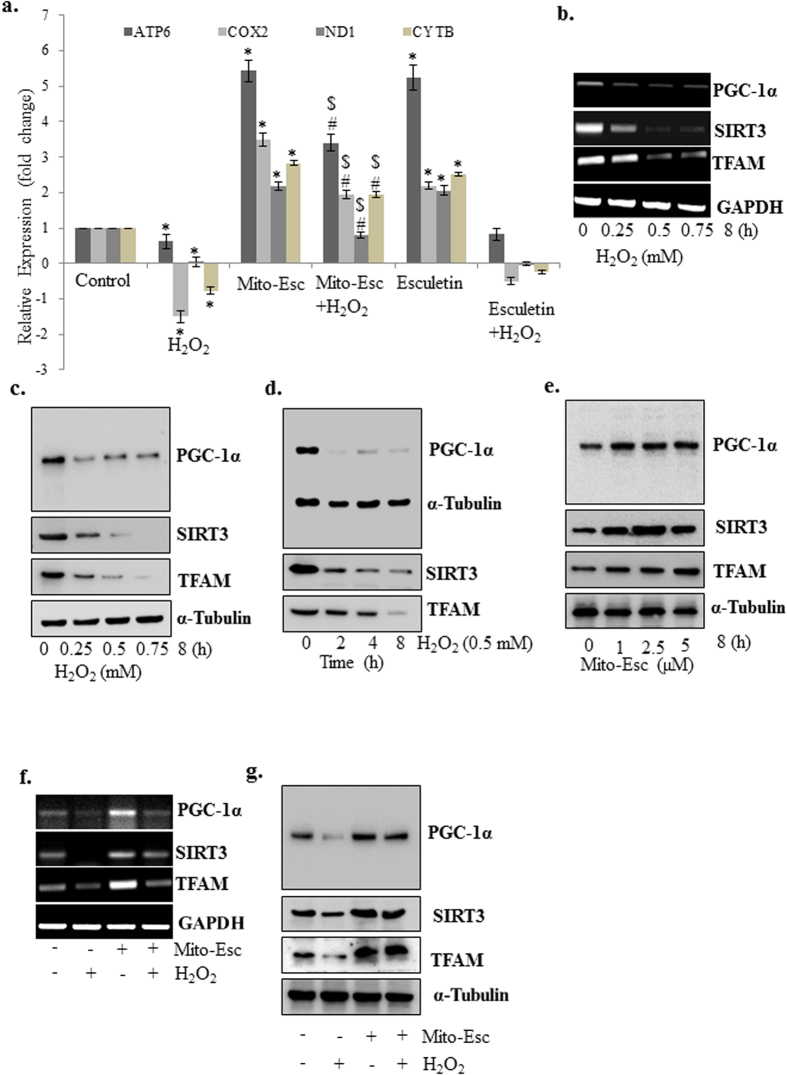
Mito-Esc pretreatment rescues oxidant-induced deregulation of mitochondrial biogenesis. (**a**) HAEC were pretreated with Mito-Esc (2.5 μM) for 2 h prior to the addition of H_2_O_2_ (0.5 mM) for 8 h and mitochondria genome-encoded gene expressions as indicated were measured by qRT-PCR. (**b**) HAEC were treated with various concentrations of H_2_O_2_ (0.25–0.75 mM) for 8 h and PGC-1α, SIRT3 and TFAM mRNA levels were measured by RT-PCR. (**c**) Same as *b* except that, protein levels were measured by Western blot analysis. (**d**) Same as *c* except that cells were incubated with H_2_O_2_ (0.5 mM) for various time points (2–8 h). (**e**) Same as *d* except that, cells were treated with different concentrations of Mito-Esc (1–5 μM) for 8 h. (**f**,**g**) HAEC were pretreated with Mito-Esc for 2 h prior to the addition of H_2_O_2_ (0.5 mM) for 8 h and PGC-1α, SIRT3 and TFAM mRNA (**f**) and protein (**g**) levels were measured by RT-PCR and Western blot analysis respectively. *Significantly different (p < 0.05) compared to untreated conditions. ^#^Significantly different (p < 0.05) compared H_2_O_2_ treated condition. Results indicated are average of three independent experiments.

Since oxidative stress is known to affect mitochondrial injury and dysfunction, we explored the effect of both Mito-Esc and natural esculetin on mitochondrial content by measuring mitochondria genome-encoded gene expressions of ATP6, COX-2, ND1, and cytochrome b. HAEC were treated with either H_2_O_2_ alone or in the presence of either Mito-Esc or natural esculetin for 8 h and qRT-PCR was performed. Interestingly, H_2_O_2_-induced loss of all the mitochondria genome-encoded gene expressions that were measured was significantly restored only in the presence of Mito-Esc but not with natural esculetin (Fig. 5a). It is to be noted that either Mito-Esc or natural esculetin treatments alone increased all these gene expressions when compared to untreated conditions (Fig. 5a). In agreement with these results, H_2_O_2_ treatment dose- and time-dependently reduced the mitochondrial biogenetic markers TFAM, PGC-1α and SIRT3 both at mRNA and protein levels (Fig. 5b–d). In contrast, Mito-Esc treatment alone as low as 1 μM, significantly elevated all these aforementioned markers (Fig. 5e). In addition, Mito-Esc greatly rescued H_2_O_2_-mediated depletion of all these markers both at mRNA and protein levels (Figs 4f and 5g). Thereby indicating that, mito-Esc rescues oxidant-induced deregulation of mitochondrial biogenesis in endothelial cells.

### Mito-Esc-induced SIRT3 expression by nitric oxide-dependent pathways via AMPKα activation causes increased mitochondrial biogenesis

**Figure 6:**
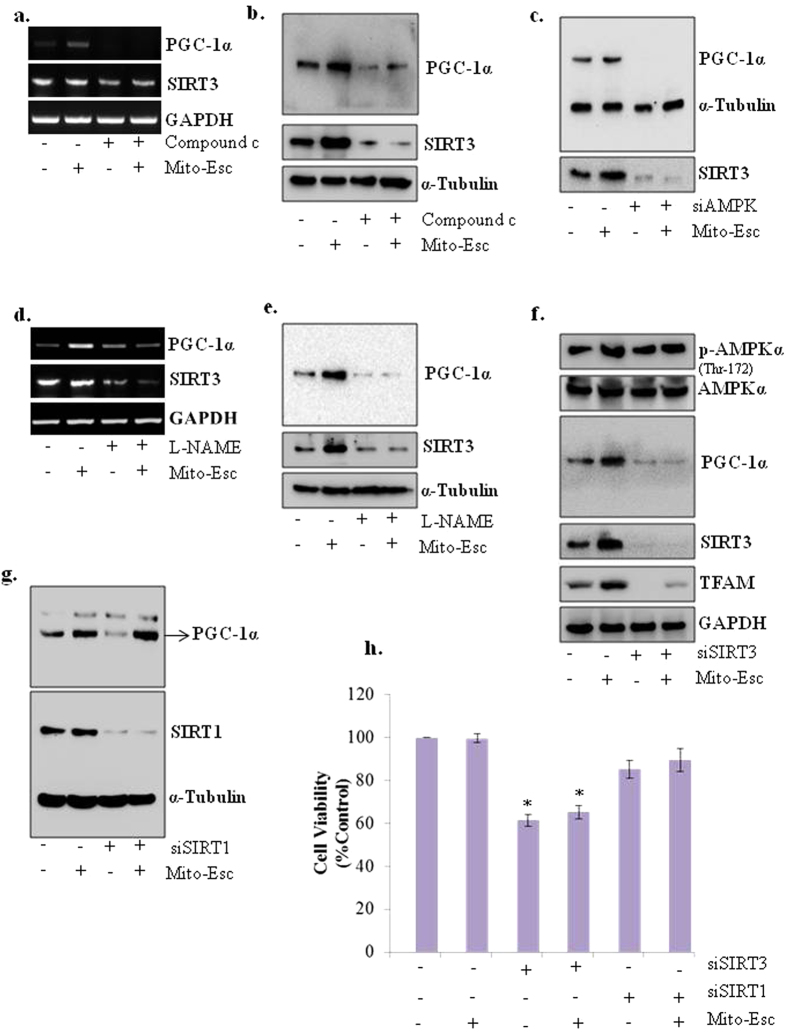
Mito-Esc-induced SIRT3 expression by AMPKα activation is responsible for increased mitochondrial biogenesis. (**a**,**b**) HAEC were pretreated with Compound c (20 μM) for 2 h prior to the addition of Mito-Esc for 8 h and then, PGC-1α and SIRT-3 transcript (**a**) and protein (**b**) levels were measured by RT-PCR and immuno blotting respectively. (**c**) same as *b* except that, cells were transfected with siAMPK for 16 h before the addition of Mito-Esc for another 8 h. (**d,e**) Cells were pretreated with L-NAME (2 mM) for 2 h prior to the addition of Mito-Esc for 8 h and then, SIRT3 and PGC-1α mRNA (**d**) and protein (**e**) levels were measured by RT-PCR and Western blot analysis respectively. (**f**) HAEC were transfected with siSIRT3 for 16 h after which, Mito-Esc was added for another 8 h and phospho-AMPKα, AMPKα, TFAM, SIRT3, and PGC-1α protein levels were measured by immunoblotting. (**g**) Same as c except that cells were transfected with siSIRT1. (**h**) Cells were transfected with either siSIRT3 or siSIRT1 for 16 h and then Mito-Esc was added for another 24 h and cell viability was measured by trypan blue assay. Results presented are average of three independent experiments. *significantly different (p < 0.05) compared to scrambled siRNA treated condition.

In this study we showed that Mito-Esc treatment to HAEC resulted in increased AMPKαphosphorylation that, in turn, caused enhanced eNOS phosphorylation and increased NO generation. In tune with these observations, we further attempted to systematically investigate the involvement of Mito-Esc-induced AMPKα and NO in regulating mitochondrial biogenesis. Treatment of cells with Compound c (AMPK inhibitor) and AMPK siRNA (siAMPK) greatly abolished Mito-Esc-induced PGC-1α and SIRT3 mRNA and protein expressions (Fig. 6a–c). Next, HAEC were incubated with L-NAME (NOS inhibitor) and found that, similar to Compound c treatment, L-NAME also significantly inhibited Mito-Esc-induced up-regulation of both PGC-1α and SIRT3 mRNA and protein expressions (Fig. 6d,e). Further, to see if Mito-Esc-induced SIRT3 is responsible for the enhanced mitochondrial biogenesis, cells were treated with siSIRT3 in the presence or absence of Mito-Esc. Interestingly, siSIRT3 completely reversed the Mito-Esc-mediated increase in PGC-1α (promotes mitochondrial biogenesis and function) and TFAM (a facilitator of mitochondrial genome transcription) (Fig. 6f). Under these conditions, however, treatment of cells with siSIRT3 failed to affect AMPKα phosphorylation (Fig. 6f). Thereby suggesting that SIRT3 regulation by Mito-Esc is downstream to AMPK activation. At this point, we were also interested to see the effect of Mito-Esc on SIRT1, another known regulator of mitochondrial biogenesis. siSIRT1 treatment although decreased PGC-1α expression, mito-Esc addition after depletion of SIRT1 by siSIRT1 still could increase PGC-1α expression (Fig. 6g). Thereby indicating that SIRT3 but not SIRT1 induction is responsible for Mito-Esc-induced mitochondrial biogenesis. Furthermore, interestingly, only siSIRT3 but not siSIRT1 treatment caused significant cell death, and moreover, incubation of cells with Mito-Esc failed to reverse the siSIRT3-induced cell death (Fig. 6h). This observation suggests that, SIRT3 plays a key role in Mito-Esc-mediated regulation of mitochondrial biogenesis and cell death.

### Mito-Esc increases Oxygen Consumption Rate (OCR) in human aortic endothelial cells

**Figure 7:**
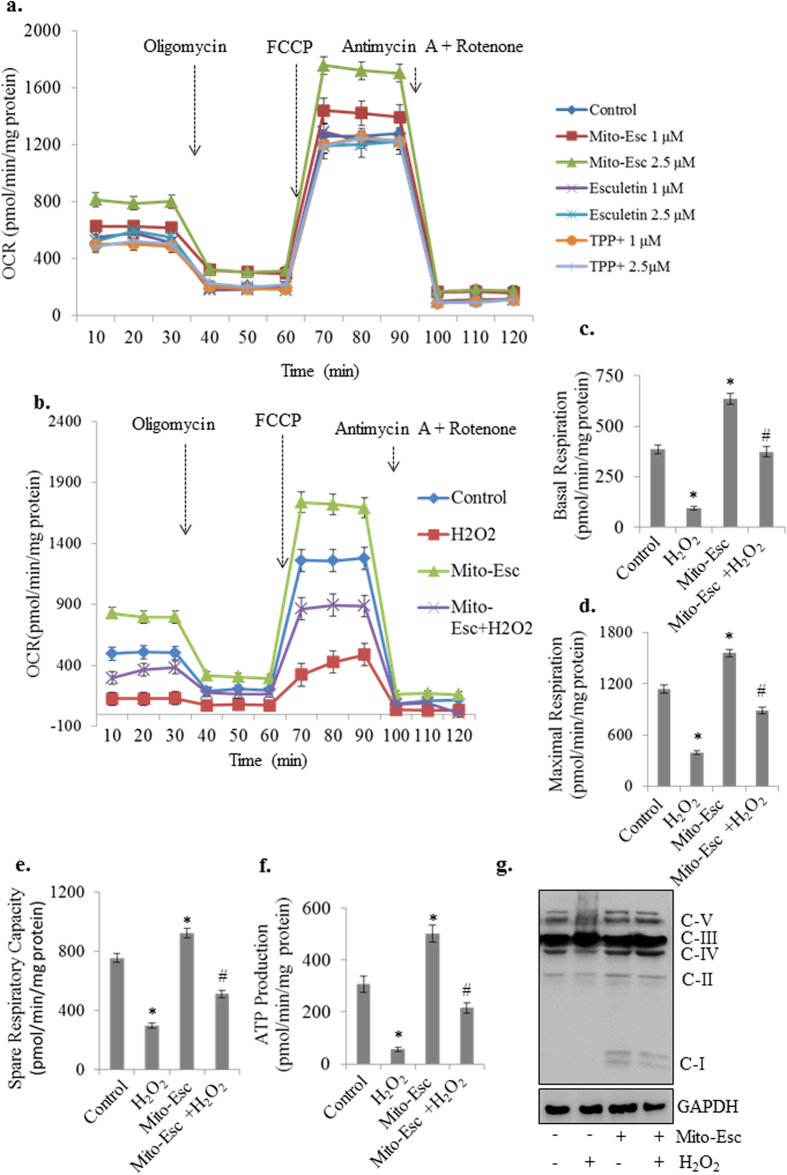
Mito-Esc but not parent esculetin rescues oxidant-induced deregulation of Oxygen Consumption Rate (OCR) in endothelial cells. (**a**) HAEC were treated with Mito-Esc, Esculetin and TPP^+^ as indicated for 6 h and oxygen consumption rate (OCR) was measured by using XF 24 Extracellular Flux Analyzer (Seahorse Bioscience). (**b**–**f**) Endothelial cells were pretreated with Mito-Esc (2.5 μM) for 2 h before H_2_O_2_ (0.5 mM) was added for another 4 h- (**b**) oxygen consumption rate (OCR) was measured by XF 24 Extracellular Flux Analyzer. Oligomycin (1 μM), FCCP (1 μM), Rotenone (1 μM) + Antimycin A (1 μM) were sequentially added (indicated by arrows) and measured the basal respiration (**c**) maximal respiration (**d**) Spare respiratory capacity (**e**) and ATP production (**f**). (**g**) Cells were pretreated with Mito-Esc (2.5 μM) for 2 h before H_2_O_2_ (0.5 mM) was added for another 4 h and the levels of OXPHOS complex subunits were measured by immunoblotting. Results presented are average of three independent experiments. *Significantly different (p < 0.05) compared to untreated conditions. ^#^Significantly different (p < 0.05) compared H_2_O_2_ treated condition.

To further understand the functional significance of Mito-Esc in promoting mitochondrial biogenesis, mitochondrial respiration was assessed using Seahorse extra cellular flux analyzer (Fig. 7a,b). Initially, when we checked the effect of either Mito-Esc or natural esculetin or TPP^+^ on OCR, it was observed that only Mito-Esc treatment but not esculetin or TPP^+^ greatly increased OCR (Fig. 7a). Next, treatment of cells with H_2_O_2_ drastically reduced the basal respiration compared to untreated conditions and interestingly, Mito-Esc pretreatment significantly rescued cells from H_2_O_2_-induced inhibition of basal respiration (Fig. 7b). Similarly, basal respiration and also the maximal respiration (induced by FCCP) were significantly enhanced by Mito-Esc treatment alone (Fig. 7c,d). Also, Mito-Esc pretreatment rescued cells from H_2_O_2_-induced dysregulation of both basal and maximal respiration (Fig. 7c,d). Mito-Esc effect also impacted on spare respiratory capacity and protected cells from H_2_O_2_-induced inhibition of respiratory function (Fig. 7e). Pretreatment of Mito-Esc restored the ATP levels which were decreased with H_2_O_2_ treatment (Fig. 7f). In agreement with these results, the mitochondrial expression of electron transport chain complexes that were inhibited with H_2_O_2_ treatment (complexes I, II, IV and V) were significantly reversed in the presence of Mito-Esc (Fig. 7g). Taken together, all these observations suggest that Mito-Esc greatly improves mitochondrial respiration and protects endothelial cells from oxidant-induced mitochondrial dysfunction.

### Mito-Esc but not natural esculetin administration attenuates the incidence of Ang-II-induced atheromatous plaque formation in ApoE^−/−^ mice

**Figure 8:**
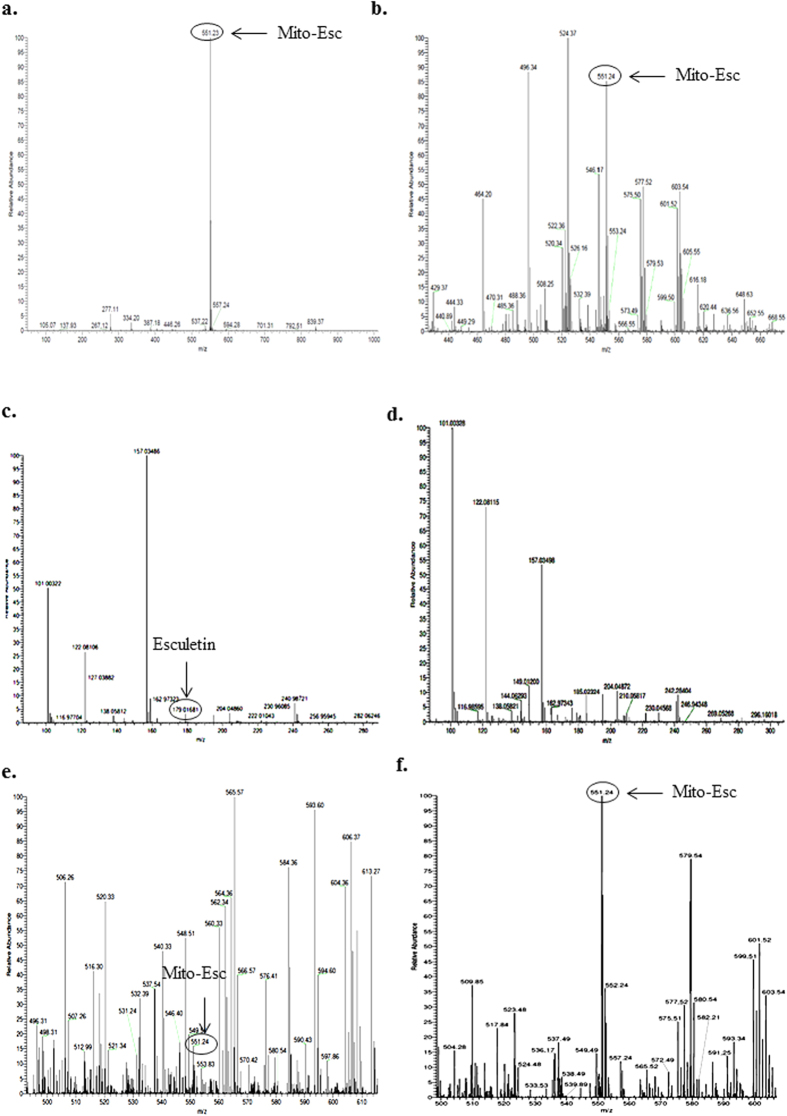
ESI-MS spectra of pure Mito-Esc (**a**), mitochondrial fraction of total aorta of Ang-II + Mito-Esc treated mice (**b**), cytosolic fraction of total aorta of Ang-II + Esculetin treated mice (**c**), mitochondrial fraction of total aorta of Ang-II + Esculetin treated mice (**d**), cytosolic fraction of HAEC treated with Mito-Esc (**e**) and mitochondrial fraction of HAEC treated with Mito-Esc (**f**) as described in methods section.

**Figure 9:**
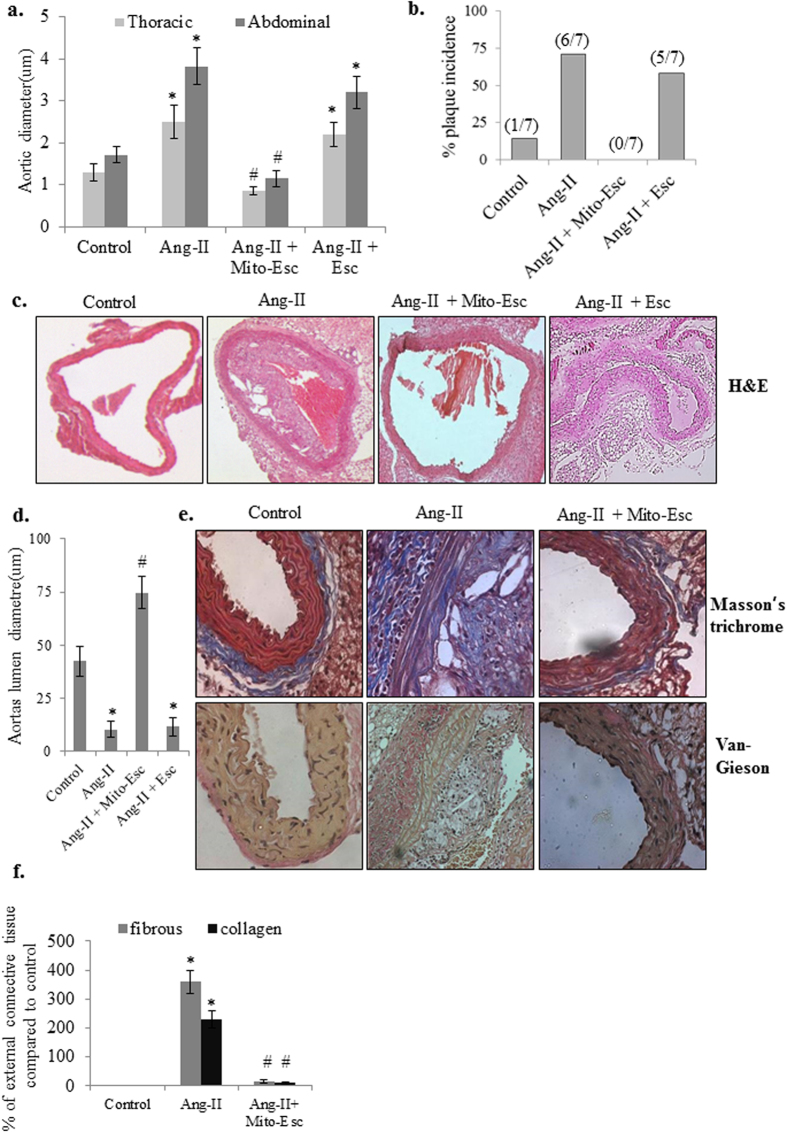
Mito-Esc administration inhibits Ang-II-induced plaque formation in ApoE^−/−^ mice. (**a**) Thoracic and abdominal aortic diameters in control, Ang-II, Ang-II + Mito-Esc and Ang-II + Esculetin treated groups. (**b**) percent plaque incidence. (**c**) Histopathological images of aorta stained with H&E. (**d**) Shows aortas lumen diameter. (**e**) Histopathological images of aortas stained with Masson trichrome and Van Gieson for analyzing fibrous and collagen tissue in the vessel wall. (**f**) Quantitative analysis of collagen and fibrous tissue in the external region of the vessel wall shown in *e*. *Significantly different (p < 0.05) compared to control group. ^#^Significantly different (p < 0.05) compared to Ang-II treated group.

It is well documented that endothelial dysfunction is the dominant risk factor for the development of vascular disorders including atherosclerosis. Concerning to this, we have investigated the physiological significance of Mito-Esc in attenuating Ang-II-induced atherogenesis in ApoE^−/−^ mice. At the end of the treatment protocol, initially, to see if mito-Esc administration has resulted in the accumulation of Mito-Esc in the aorta, we have fractionated the aortic homogenate into cytosolic and mitochondrial extracts and measured the Mito-Esc levels using ESI-mass spectrometry. Interestingly, we could detect only Mito-Esc but not parent esculetin in the mitochondrial fraction of the aorta (Table 1 and Fig. 8a–d). In agreement with this result, an increased accumulation of Mito-Esc but not parent esculetin was observed in the mitochondrial fraction of HAEC (Table 1 and Fig. 8e,f). Also, the thoracic and abdominal aorta of Ang-II + Mito-Esc treated group showed a significant reduction in Ang-II-induced a) maximal aortic diameters, b) plaque extension at the end of six weeks (Fig. 9a,b). These changes in Ang-II + Mito-Esc group were comparable to control group mice.

**Figure 10:**
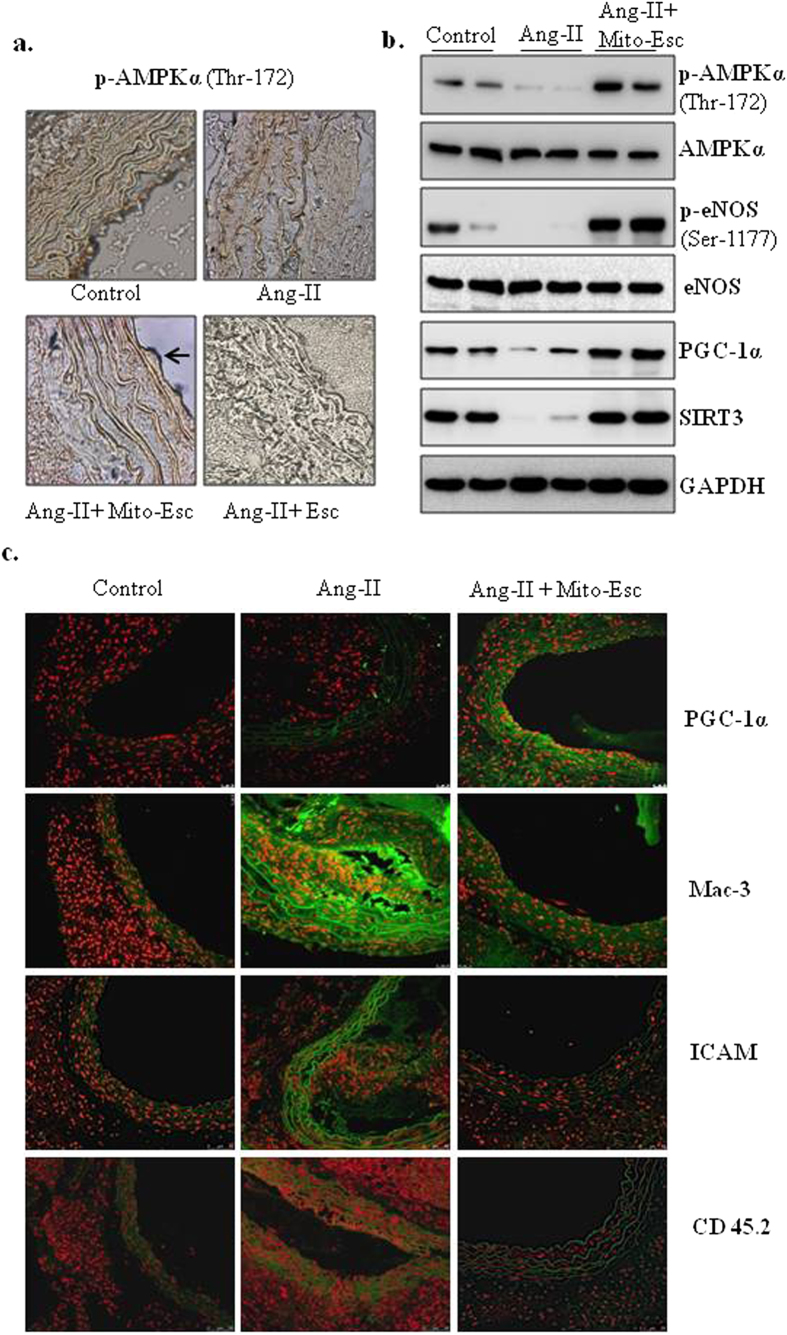
Mito-Esc administration rescues Ang-II-induced alterations in phospho-AMPK, phospho-eNOS, PGC-1α, monocyte infiltration and inflammatory markers in the aorta. (**a**) Shows the phospho-AMPKα levels by Immunohistochemistry. (**b**) Represents phospho-AMPKα, AMPKα, phospho-eNOS, eNOS, PGC-1α and SIRT3 protein levels measured in the aortic tissue homogenate by immunoblotting. Quantification of *b* is presented in supplementary Fig. S2. (**c**) Shows the PGC-1α, Mac-3, ICAM and CD45.2 immunofluorescence (green fluorescence represents positive staining as indicated) by confocal microscopy.

We further analyzed the vascular remodeling by using histological stains in tissue sections of thoracic aortas. H&E staining of Ang-II + Mito-Esc but not Ang-II + Esculetin treated group aorta showed a complete protection from Ang-II treatment-induced atherosclerotic lesions and intimal plaque formation (Fig. 9c,d). Masson’s trichrome staining revealed thick fibrous mature connective tissue surrounding/in between atheroma in Ang-II treated mice aorta which was significantly reduced in Ang-II + Mito-Esc treated group (Fig. 9e,f). The collagen tissue in the atheroma, intimal, medial and external region appeared as blue color indicative of extensive proliferation of collagen tissue in the atheromatous region of Ang-II treated mice. However, in control and Mito-Esc treated groups, only marginal collagen tissue was observed. Also, Van Gieson staining indicated ruptured medial layer lamella along with the dark brown color nucleus of the lamella in Ang-II treated mice (Fig. 9e). To further corroborate Mito-Esc’s ability to protect from Ang-II-induced endothelial dysfunction during the progression of atheromatous plaque formation, phospho-eNOS, eNOS, phospho-AMPKα and AMPKα protein levels were measured by immunoblotting in total aortic tissue homogenate and also by immunohistochemistry. Interestingly and in agreement with endothelial cell culture results, endothelial cell lining of the aorta showed increased staining of phospho-AMPKα in Ang-II + Mito-Esc administered groups when compared to Ang-II alone administered group (Fig. 10a). Similar results were seen even by immunoblotting (Fig. 10b and Supplementary Fig. S2). Also, phospho-eNOS and PGC-1α levels were significantly increased in Ang-II + Mito-Esc administered group compared to Ang-II alone treated group (Fig. 10b,c and Supplementary Fig. S2). Similarly, SIRT3 protein levels were found to be more in Ang-II + Mito-Esc administered group compared to either Ang-II alone or control groups (Fig. 10b and Supplementary Fig. S2). This suggests that, Mito-Esc by increasing eNOS-derived nitric oxide generation restores endothelial function in Ang-II administered ApoE^−/−^ mice. These results are in agreement with cell culture results, wherein, Mito-Esc treatment greatly increased the phosphorylations of both eNOS and AMPKα along with the mitochondrial biogenetic markers in HAEC (Figs 4, 5, 6). We have also measured Mac-3 levels by immunofluorescence. Mac-3 is a general marker for macrophage abundance often seen under inflammatory conditions. Ang-II treatment greatly elevated Mac-3 levels indicating an increased macrophage accumulation in the atheromatous region (Fig. 10d). Similarly, increased levels of ICAM and CD45.2 by immunofluorescence were observed in Ang-II alone treated group (Fig. 10c). In contrast, Ang-II + Mito-Esc group showed an inhibition of Mac-3 along with ICAM and CD45.2 stainings (Fig. 10c). These results indicate that Mito-Esc administration significantly inhibits Ang-II-induced triggering of inflammatory cascades in the atheromatous region of the aorta. In tune with this, Ang-II + Mito-Esc treated mice showed a significant inhibition of Ang-II-induced-proinflammatory cytokines (TNF-α, IFN- **γ**, MCP-1) production (Supplementary Fig. S3). Finally, to extend the vasculoprotective effects of Mito-Esc, it was observed that, Mito-Esc treatment significantly reduced Ang-II-mediated increase in LDL, VLDL, triglycerides and total cholesterol (Supplementary Table S1). Also importantly, Mito-Esc treatment resulted in a significant rise in serum HDL levels (Supplementary Table S1). Taken together, all these results implicate that Mito-Esc treatment significantly eases the incidence of vascular complications including plaque formation.

## Discussion

Endothelial dysfunction is one of the most common contributing factors for the development and progression of vascular diseases and that mitochondrial functional alteration because of enhanced oxidant generation are implicated in vascular endothelial dysfunction. In the present study, we explored the therapeutic potential of mitochondrially-targeted esculetin in mitigating oxidant-induced endothelial cell death and subsequently its effect in regressing Ang-II-induced atherosclerosis in ApoE^−/−^ mice. The results showed that Mito-Esc but not natural esculetin significantly protected endothelial cells from H_2_O_2_- and Ang-II-induced cell death. This presumably was due to the relatively low bioavailability of esculetin when compared to Mito-Esc at concentrations (2.5 μM) that were employed in this study. Moreover, we were unable to detect natural esculetin in the mitochondrial fraction, while Mito-Esc significantly accumulated in the mitochondria by ESI-MS. These results are in line with the inhibition of mitochondrial superoxide generation by Mito-Esc but not esculetin during H_2_O_2_-induced oxidative stress. It is reasonably known that enhanced generation of mitochondria-derived oxidants plays a major role in inducing endothelial dysfunction^22^. It was shown that Ang-II by increasing H_2_O_2_ production causes endothelial dysfunction^22^. In support of this, in the present study, endothelial cells treated with Ang-II increased H_2_O_2_ and mitochondrial superoxide production. While understanding the mechanisms responsible for Mito-Esc mediated protective effects during oxidant-induced endothelial cell death, it was observed that Mito-Esc but not natural esculetin greatly enhanced nitric oxide production via increased phosphorylation of eNOS. Nitric oxide levels were significantly reduced with either H_2_O_2_ or Ang-II treatment in HAEC. Interestingly, treatment of cells with L-NAME (NOS inhibitor), significantly reversed Mito-Esc mediated protective effect during oxidative stress. Thereby suggesting that Mito-Esc protects against oxidant-induced endothelial cell damage because of its ability to increase NO levels and this, in part, is attributed to its anti-apoptotic effects. Previously we have shown that nitric oxide by scavenging peroxyl radicals; inhibit oxidized-LDL-induced apoptosis in endothelial cells^28^.

It is well established that NO plays a pivotal role in maintaining vascular homeostasis, and its levels are compromised during vascular abnormalities including atherosclerosis, as a result of endothelial dysfunction^28^,^29^. Mitochondria-targeted ubiquinone (MitoQ) by increasing NO bioavailability completely restored endothelium-dependent dilation (EDD) in old mice^30^. One of the important observations of the present study was that Mito-Esc treatment significantly increased AMPK activation and this in turn was responsible for increased phosphorylation of eNOS, thereby resulting in enhanced NO levels as inhibition of AMPK activity by Compound c significantly hampered Mito-Esc-induced NO generation in endothelial cells. These observations were further corroborated under *in vivo* conditions wherein, Mito-Esc administration for 6 weeks caused a significant upregulation of both AMPKα and eNOS phosphorylations in Ang-II + Mito-Esc administered group compared to Ang-II alone administered group. In support of the observed favourable effects of this newly synthesized mitochondria-targeted esculetin, we also noticed a complete regression of Ang-II-induced atheromatic plaque formation in Mito-Esc but not in natural esculetin administered mice. Intriguingly, we were able to detect Mito-Esc in the mitochondrial fraction of the aorta by ESI-MS. Therefore, it is plausible that the protective effects elicited by Mito-Esc were due to its presence in the mitochondria of vascular cells. In atherosclerosis-associated hemodialysis, diabetes, and smoking, excessive mitochondrial-ROS is observed and has been linked to a marked decrease in SOD and GPx levels^31^. In another study, it was shown that athero-prone regions of the aorta in *Sod*^+*/*−^ *ApoE*^+*/*−^ mice have accelerated atherosclerosis, mitochondrial DNA damage and accumulation of 3-nitrosylated proteins by inducing mitochondrial oxidative stress^32^. Atherosclerotic lesions in human and rabbit were shown to have high levels of oxidized cardiolipin, a phospholipid exclusively expressed in mitochondria, thereby suggesting enhanced mitochondrial-ROS generation^33^. The failure of intracellular anti-oxidative defence mechanisms during the progression of atherosclerosis was in part attributed to the peroxynitrite-mediated inactivation of MnSOD^34^. Interestingly, it was found that Mito-Esc not only promoted mitochondrial biogenesis, but also rescued endothelial cells from oxidant-induced mitochondrial deregulation. Importantly, Mito-Esc by inducing mitochondrial biogenesis functionally enhanced the mitochondrial respiration by elevating the basal and maximal respiration along with the spare respiratory capacity and ATP production. All these parameters presumably ease the burden on the mitochondrial bioenergetics especially during oxidative insults on the cell. From the results obtained in the present study, it appears that Mito-Esc-induced AMPKα phosphorylation is independent of ATP levels, as Mito-Esc treatment increased both AMPKα phosphorylation and ATP levels in HAEC. It is now reasonably known that SIRT3 is involved in mitochondrial biogenesis^35^,^36^. In the present study, we show that Mito-Esc-induced mitochondrial biogenesis and function are predominantly mediated by the induction of SIRT3, as silencing of SIRT3 completely abrogated the Mito-Esc-mediated effects on mitochondrial biogenesis. Under the same conditions, Mito-Esc treatment failed to affect siSIRT3 induced endothelial cell death. Thereby implicating the key role played by SIRT3 in maintaining the endothelial cell survival. Previously it was shown that SIRT3 by deacetylating MnSOD (mitochondrial SOD), increases its activity, which in turn causes the dismutation of superoxide radicals^37^,^38^,^39^. In line with this, in the present study, mitochondrial superoxide levels were significantly inhibited by the Mito-Esc treatment. However, at this point, we do not know whether the inhibition of mitochondrial superoxide by Mito-Esc treatment is because of its ability to induce SIRT3 activation or because of its direct superoxide scavenging effect or both.

Also in the present study, the gross pathological observations in Ang-II + Mito-Esc treated mice indicated extensive plaque reduction in thoracic and abdominal aorta when compared Ang-II alone treated mice. Along with this, Mito-Esc administered mice aorta showed the significant reduction in monocyte/macrophage infiltration, ICAM and CD45.2 levels suggesting that, Mito-Esc perturbs Ang-II-induced inflammatory cascades during the progression of atheroma formation.

One of the other important observations of this study, was that Mito-Esc administration significantly inhibited Ang-II-induced serum LDL and triglyceride levels while the levels of HDL were increased. Along these lines, Mito-Esc greatly inhibited the Ang-II-mediated increase in proinflammatory cytokines production. Recently it was shown that specific inhibition of mitochondrial oxidative stress by mitochondria-targeted vitamin E reduced proinflammatory cytokines levels and improved cardiac function in a mouse sepsis model^40^. In conclusion, we have synthesized a novel mitochondria-targeted esculetin (Mito-Esc), and the present study provides new insights into the vasculoprotective effects of Mito-Esc by enhancing a) AMPK-mediated nitric oxide generation via increased eNOS phosphorylation and b) mitochondrial biogenesis through increased SIRT3 expression. Furthermore, Mito-Esc treatment attenuated angiotensin-II-induced plaque formation, potentially by affecting mitochondrial ROS, mitochondrial biogenesis and inflammatory pathways in ApoE^−/−^ mice.

## Experimental procedures

### Endothelial cell culture

Human aortic endothelial cells (HAECs) were obtained from ATCC (Manassas, VA) and maintained (37 °C, 5% CO_2_) in basal medium supplemented with 10% FBS and growth supplements (EBM-2 bullet kit, Lonza). Cells used in this study were between passages 4 and 9.

### Trypan blue cell viability assay

At the end of the treatments, cells were harvested and re-suspended in 0.4% trypan blue and percent cell viability^41^ was counted using countess cell chamber (Life Technologies).

### Caspase-3 and -8 activities

Caspase-3 and -8 activities were measured according to a previously published protocol^42^.

### Measurement of H_2_O_2_ levels

Amplex Red reagent was used to detect the released H_2_O_2_ from cells. At the end of the treatments, HAECs were trypsinized, and 20,000 cells were resuspended in 100 μl of Kreb’s Ringer phosphate buffer (pH, 7.35) and the assay was initiated by mixing with 100 μl of KRB solution containing 50 μM Amplex Red reagent along with 0.1 U/mL HRP. Immediately, the formation of resorufin fluorescence was measured in a multimode reader at λex = 540 nm and λem = 585 nm^23^.

### Measurement of GSH levels

This was followed according to the method developed by Hissin PJ and Hilf R^43^.

### Detection of mitochondrial transmembrane potential changes

Mitochondrial membrane potential was measured by using tetramethylrhodamine ethyl ester (TMRE-) as described^44^.

### Detection of mitochondrial superoxide

HAEC were treated with H_2_O_2_ in the presence or absence of Mito-Esc or parent esculetin for 8 h. Cells were washed free of medium and incubated with 10 μM Mito-SOX for 20 min and fluorescence images were captured using Olympus fluorescence microscope equipped with Rhodamine filter^45^.

### Measurement of intracellular nitric oxide (NO) levels

Intracellular NO levels were monitored by using the DAF-2DA fluorescence probe and by estimating the nitrite levels as described previously^29^.

### Western blot analysis

At the end of the treatments, proteins were resolved by SDS-PAGE and blotted onto nitrocellulose membrane and probed with rabbit anti-phospho-eNOS (ser-1177), rabbit anti-eNOS, rabbit anti-phospho-AMPKα (Thr-172), rabbit anti-AMPKα, PGC-1α, SIRT3, TFAM and GAPDH, Mouse-anti-SIRT1 and α-Tubulin antibodies, and then incubated with horseradish peroxidase-conjugated goat anti-rabbit IgG or goat-anti-mouse IgG secondary antibody(1:5000). Protein bands were detected by using HRP substrate. All the antibodies used in this study were from cell signaling Technologies, except PGC-1α, TFAM and SIRT1.

### Gene expression studies using RT-PCR

Total RNA was isolated using TRIzol (Sigma) reagent. Subsequently, cDNA was prepared using 1 μg RNA according to the manufacturer’s instructions (Fermentas cDNA synthesis kit). The indicated genes were amplified using gene specific primers.

### Analysis of mitochondrial respiration

An extracellular flux analyzer (XF24: Seahorse Biosciences) was used to analyze the mitochondrial function. HAECs were seeded at a density of 40,000 cells per well in EBM-2 media. After 12 h, cells were treated with Mito-Esc for 2 h and H_2_O_2_ (wherever applicable) was added after two hours of Mito-Esc treatment and continued for another 4 h. EBM-2 media was replaced with Seahorse media containing 25 mM glucose and incubated for 1 h at 37 °C in a CO_2_ free incubator for equilibration. Oligomycin, FCCP, Antimycin A + Rotenone were preloaded in Ports-A, B and C respectively in the reagent ports. Oxygen consumption was obtained from the slopes of concentration change *verses* time. Maximal respiration was calculated by FCCP-induced oxygen consumption rate minus the Rotenone + Antimycin A inhibited oxygen consumption rate. Spare respiratory capacity was calculated by FCCP-induced oxygen consumption rate minus oxygen consumption rate at base line. ATP production was calculated by the base line oxygen consumption rate minus oligomycin inhibited oxygen consumption rate^46^.

### Detection and quantification of Mito-Esc by Mass Spectrometry

Initially, mitochondrial and cytosolic fractions were separated using a commercially available kit (ProteoExtract Cytosol/Mitochondria Fractionation Kit, Merck, USA) according to manufacturer’s instructions. Mito-Esc was quantified in the mitochondrial and cytosolic fractions obtained from HAEC and aortas of ApoE^−/−^ mice of different treatment groups as mentioned in the Animal Experiments section. Electrospray ionization (ESI)-mass spectrometry (MS) measurements (positive mode) were performed using a quadrupole time-of-flight mass spectrometer (QSTAR XL, Applied Biosystems/MDS Sciex, Foster City, CA). The data acquisition was under the control of Analyst QS software (Applied Biosystems). For the CID (collision-induced dissociation) experiments, the precursor ions were selected using the quadrupole analyzer and the product ions were analyzed using the TOF analyzer^47^.

The mitochondrial/cytosolic extracts (50 μl) were diluted with 50 μl of methanol and introduced into the ESI source (injection volume 20 μl) using methanol: water (80:20, v/v) as a mobile phase gradient with flow rate 600 μl/min. Stock solution (0.5 mM) of Mito-Esc was made in methanol: water (50:50, v/v). For spiking experiments, appropriate volumes of standard solutions (1–50 μM) were added to the mitochondrial/cytosolic extracts of untreated samples.

### Animal experiments

Experiments were conducted in two months old male apolipoprotein E knockout (ApoE^−/−^) mice according to the guidelines formulated for care and use of animals in scientific research (ICMR, India) at a CPCSEA (Committee for the Purpose of Control and Supervision of Experiments on Animals) registered animal facility.The experimental protocols were approved by the IAEC at CSIR-IICT (IICT/CB/SK/20/12/2013/10). Animals were randomly divided into 3 groups each *n* = *7*; 1) control 2) Ang-II treatment and 3) Mito-Esc + Ang-II treatment. Ang-II and the Mito-Esc treatment groups received Ang-II (Sigma) at a dose of 1.44 mg/kg/day as described previously^48^,^49^,^50^ for 6 weeks through sub-cutaneous route whereas the control group received normal saline. Mito-Esc treatment group received the compound at a dose of 0.5 mg/kg/day in normal drinking water. All the animals were fed on normal chow throughout the study. After 6 weeks, animals were sacrificed as per the standard protocols by euthanasia.

### Histopathological analysis

Aortas were excised and measured the thoracic and abdominal diameters using vernier caliper. For immunohistochemistry, thoracic aortas were fixed with 10% buffered formalin and processed for paraffin embedding. Serial sections of 5 μm thickness were made and stained as described previously^50^,^51^,^52^.

### Analysis of serum lipids and cytokine levels

Before euthanasia, blood was collected by orbital sinus puncture under isoflurane-induced anesthesia, and serum was separated. Total cholesterol (TC), HDL, LDL, VLDL and triglyceride (TG) were assayed using commercial kits (Coral Clinical Systems, The Tulip Group, India). Serum cytokines levels were measured using BD multiplex assay kits according to Manufacturer’s instructions.

### Statistical analysis

Data were expressed as mean ± SD. They were statistically analyzed by the two-tailed, unpaired, Student’s t-test and scores were considered significant at P < 0.05.

## Additional Information

**How to cite this article**: Karnewar, S. *et al*. Mitochondria-targeted esculetin alleviates mitochondrial dysfunction by AMPK-mediated nitric oxide and SIRT3 regulation in endothelial cells: potential implications in atherosclerosis. *Sci. Rep.* **6**, 24108; doi: 10.1038/srep24108 (2016).

## Supplementary Material

Supplementary Information

## Figures and Tables

**Table 1:** Cellular uptake of Esculetin and Mito-Esc.

**Condition**	**Cytosolic fraction (nmol**/**mg protein)**	**Mitochondrial fraction (nmol**/**mg protein)**
Esculetin (HAEC)	6249 ± 235	ND
Mito-Esc (HAEC)	14523 ± 342	4488 ± 104
Esculetin + Ang-II (Apo E^−/−^ mice aorta)	1826 ± 234	ND
Mito-Esc + Ang-II (Apo E^−/−^ mice aorta)	ND	2547 ± 286
