# Supplementary material for: Mitochondria-targeted esculetin alleviates mitochondrial dysfunction by AMPK-mediated nitric oxide and SIRT3 regulation in endothelial cells: potential implications in atherosclerosis
Source: Sci Rep. 2016 Apr 11;6:24108. doi: 10.1038/srep24108 (PMC4827087; doi:10.1038/srep24108)
Supplement: Supplementary Information [file srep24108-s1.pdf]

Mitochondria-targeted esculetin alleviates mitochondrial dysfunction by AMPK-mediated nitric oxide and SIRT3 regulation in endothelial cells: potential implications in atherosclerosis

Santosh Karnewar[1,2], Sathish Babu Vasamsetti[1,2], Raja Gopoju[1,2], Anantha Koteswararao Kanugula[1], Sai Krishna Ganji[3], Sripadi Prabhakar [3], Nandini Rangaraj[4], Nitin Tupperwar[4] , Jerald Mahesh Kumar[4]* and Srigiridhar Kotamraju[1,2]*

[1]Centre for Chemical Biology, CSIR-Indian Institute of Chemical Technology, Uppal Road, Hyderabad-500007, India. [2]Academy of Scientific and Innovative Research, Training and Development Complex, Chennai, India. [3]National Centre for Mass Spectrometry, Indian Institute of Chemical Technology, Hyderabad-500007, India. [4]CSIR-Centre for Cellular and Molecular Biology, Uppal Road, Hyderabad-500007, India.

Correspondence:
Dr. Srigiridhar Kotamraju, Centre for Chemical Biology, CSIR-Indian Institute of Chemical Technology, Hyderabad-500007, India. +91-40-27191867; Email: giridhar@iict.res.in
Dr. Jerald Mahesh Kumar, CSIR-Centre for Cellular and Molecular Biology, Uppal Road, Hyderabad-500007, India. +91-40-27192872; Email: mahesh73@ccmb.res.in

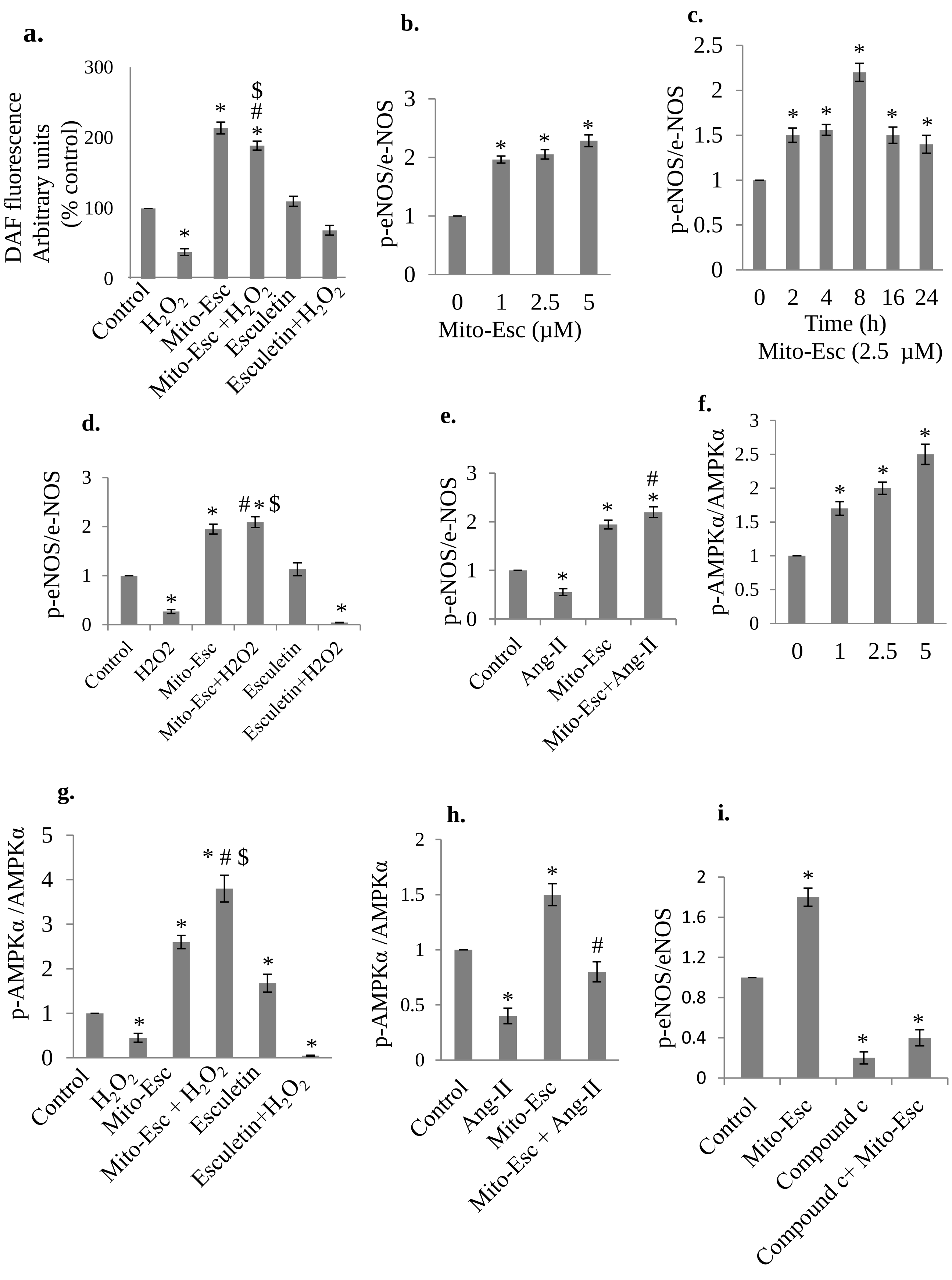

Fig. S1. Densitometric analysis of the DAF-2DA and Western blots presented in Fig.**4.**
*, significantly different ($p<0.05$) compared to control group. #, significantly different ($p<0.05$) compared to Ang-II/H_2O_2 treated group.$, significantly different compared to Esc + H_2O_2 treated group.

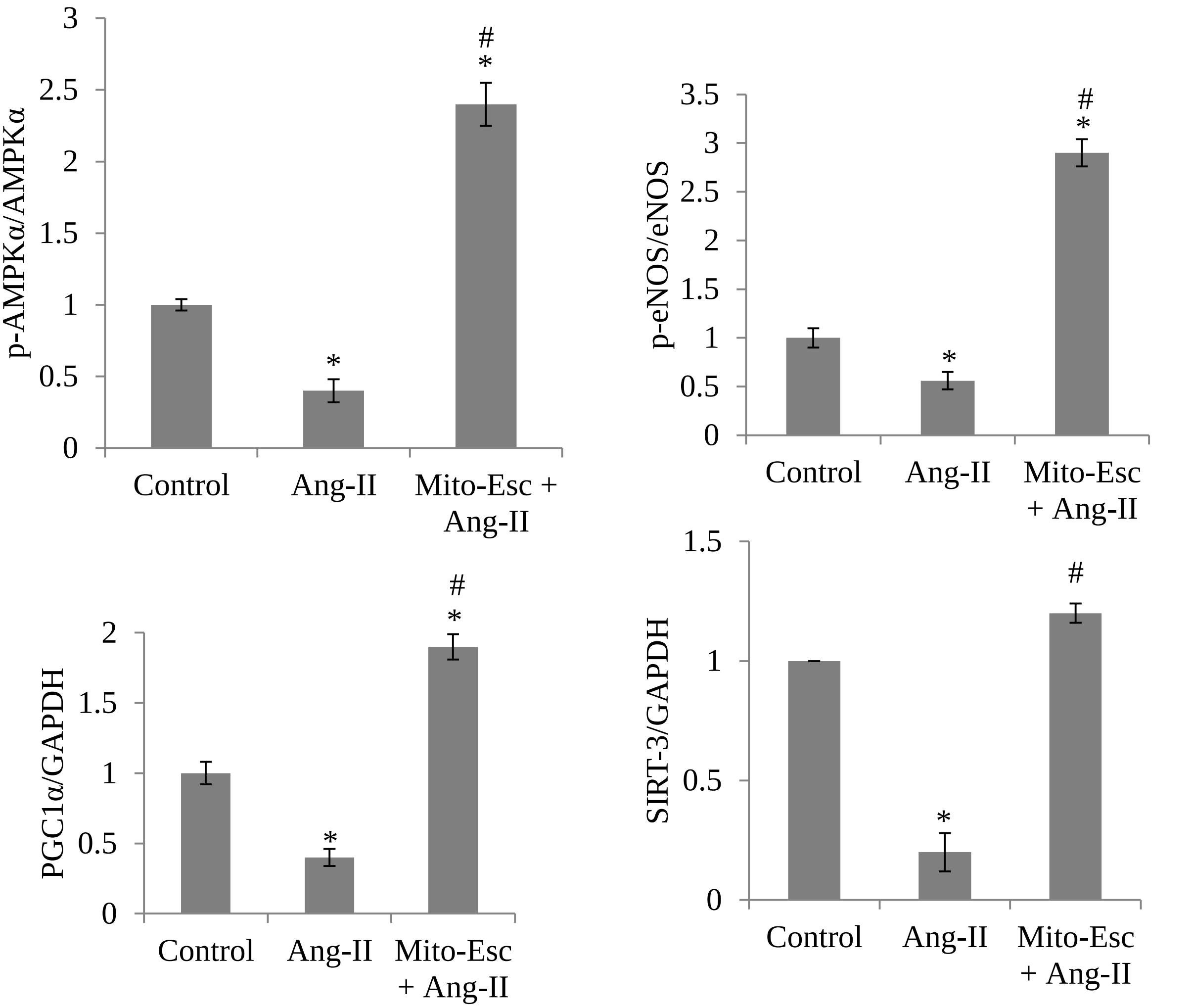

Fig. S2. Densitometric analysis of the Western blots presented in Fig.**10b**
*, significantly different ($p<0.05$) compared to control group. #, significantly different ($p<0.05$) compared to Ang-II treated group.

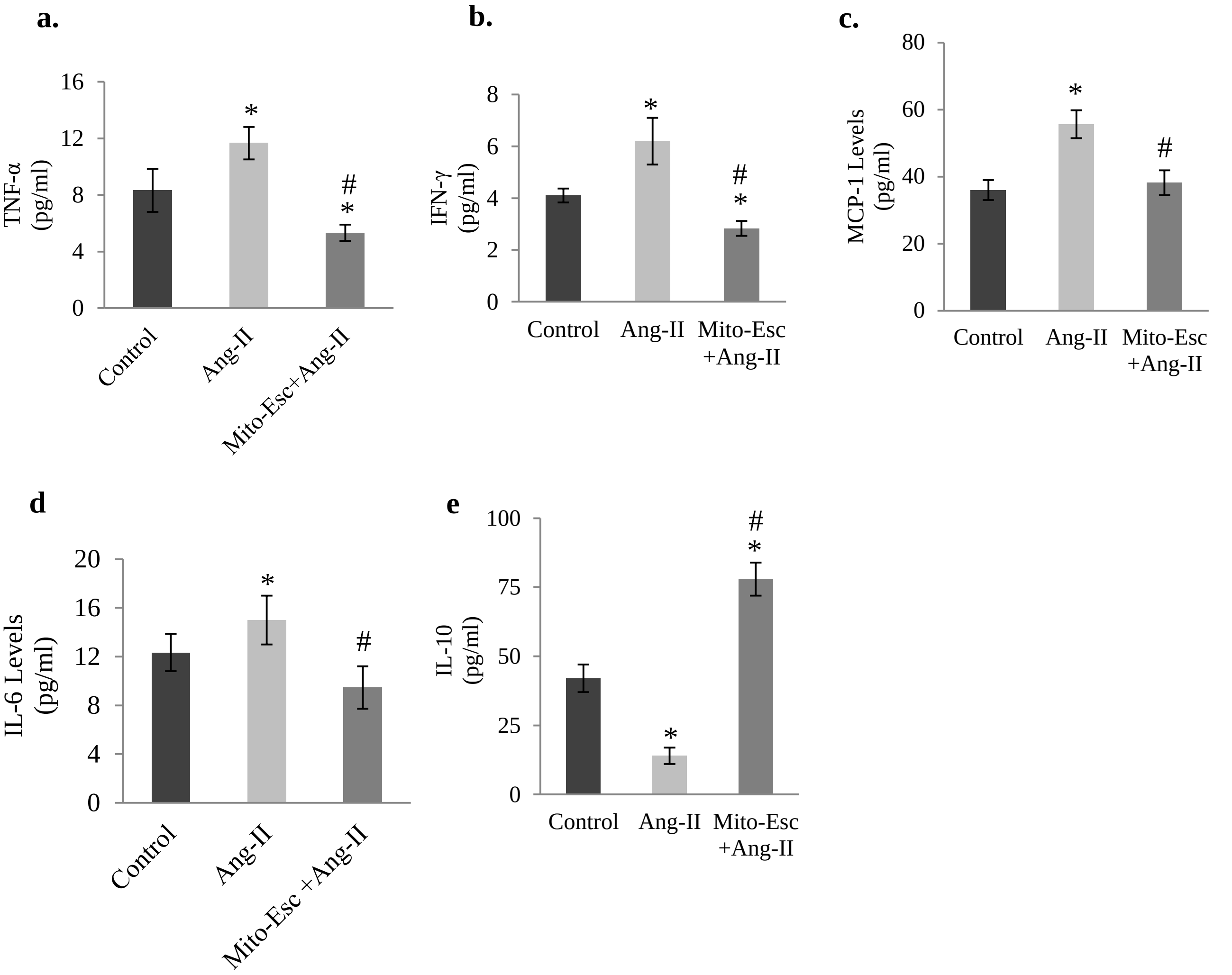

Fig.S3. Mito-Esc administration reduces Ang-II-induced proinflammatory cytokines production: (a-e) Serum cytokines levels were measured using BD multiplex assay kits according to Manufacturer's instructions. *, significantly different ($p<0.05$) compared to control group. #, significantly different ($p<0.05$) compared to Ang-II treated group.

Table S1. Alterations in lipid profile.

condition	Triglycerides (mg/dL)	LDL (mg/dL)	HDL (mg/dL)	VLDL (mg/dL)	Total Cholesterol (mg/dL)
Control	175±6	165±15	23±1.5	35±0.7	364±14
Ang-II	234±21*	423±29*	2.2±1.2*	47±2.2*	656±34*
Ang-II+ Mito-Esc	150±4#	218±21#	45±0.4#	30±0.4#	414±8#
Ang-II+ Esculetin	239±31*	409±91*	4.2±0.9*	49±3.1*	601±18*

*, significantly different (p<0.05) compared to untreated conditions. #, significantly different (p<0.05) compared to Ang-II treated group.
